# Constructing and validating nomograms to predict risk and prognostic factors of distant metastasis in urothelial bladder cancer patients: a population-based retrospective study

**DOI:** 10.1186/s12894-022-01166-6

**Published:** 2022-12-27

**Authors:** Di Chen, Zhihua Luo, Chaoping Ye, Quanhai Luo, Wenji Fan, Changsheng Chen, Gang Liu

**Affiliations:** 1 Department of Urology and Reproductive Andrology, The Reproductive Hospital of Guangxi Zhuang Autonomous Region, Nanning, 530021 Guangxi China; 2 Department of Urology and Reproductive Andrology, The Nanxishan Hospital, Guilin, Guangxi China; 3 Department of Health Management, The People’s Hospital of Guangxi Zhuang Autonomous Region and Research Center of Health Management, Guangxi Academy of Medical Sciences, Nanning, 530021 Guangxi China; 4 Department of Urology andrology, The Nanning Second People’s Hospital, Nanning, 530021 China; 5 Department of Urology, Research Center of Health Management, The People’s Hospital of Guangxi Zhuang Autonomous Region and Guangxi Academy of Medical Sciences, Nanning, 530021 Guangxi China

**Keywords:** Urothelial bladder cancer, Distant metastasis, Nomogram, Prognosis and diagnostic prediction

## Abstract

**Background:**

Urothelial carcinoma is the most common type of bladder cancer worldwide and it has a poor prognosis for patients with distant metastasis. Nomograms are frequently used in clinical research, but no research has evaluated the diagnostic and prognostic factors of distant metastasis in urothelial bladder cancer (UBC).

**Methods:**

The Surveillance, Epidemiology, and End Results database was used to analyze all patients diagnosed with UBC between 2000 and 2017. Lasso regression was used to identify the potential risk predictive factors for distant metastasis in UBC. Univariate and multivariate Cox proportional hazard regression analyses were performed to determine independent prognostic factors for distant metastasis urothelial bladder cancer (DMUBC). Subsequently, two nomograms were constructed based on the above models. The receiver operating characteristic (ROC), and calibration curves were performed to evaluate the two nomograms.

**Results:**

The study included 73,264 patients with UBC, with 2,129 (2.9%) having distant metastasis at the time of diagnosis. In the diagnostic model, tumor size, histologic type, and stage N and T were all important risk predictive factors for distant metastasis of UBC. In the prognostic model, age, tumor size, surgery, and chemotherapy were independent factors affecting the prognosis of DMUBC. DCA, ROC, calibration, and Kaplan–Meier (K–M) survival curves reveal that the two nomograms can effectively predict the diagnosis and prognosis of DMUBC.

**Conclusion:**

The developed nomograms are practical methods for predicting the occurrence risk and prognosis of distant metastasis urothelial bladder cancer patients, which may benefit the clinical decision-making process.

## Introduction

The second-highest prevailing urological malignant tumor is bladder cancer, which is also the tenth most common cancer worldwide and the seventh most prevalent tumor in men [1, 2]. According to global cancer statistics for the year 2020, there will be 573,000 new cases of bladder cancer and 213,000 deaths, resulting in a substantial financial and healthcare burden for society [1, 3, 4]. Although surgery and chemotherapy offer symptomatic relief and effective improvement in overall survival, the high recurrence rates and metastasis result in five-year survival rate of approximately 50–60%[5]. Notably, bladder cancer distant metastases are more common than recurrences, occurring in 10–15% of patients at the time of diagnosis[5].

Uroepithelium, the bladder’s inner lining, is mainly composed of urothelial cell, which are responsible for 90% of all bladder cancers [6]. Additionally, the lymph node is one of the main routes of metastasis in urothelial bladder cancer (UBC), and liver metastases have a poor prognosis [7]. Even though radical cystectomy and pelvic lymph node dissection are the current gold-standard treatment for muscle-invasive bladder cancer, over 50% of patients will eventually develop a distant micro-metastatic [8]. In particular, the treatment and prognosis of bladder cancer have changed insignificantly over the past three decades; cisplatin-based chemotherapy is still the first-line treatment for metastatic bladder cancer, but the median overall survival hardly exceeds 3–6 months [9]. Therefore, to improve treatment efficacy and prognosis, further investigation into the related factors of metastasis in urothelial bladder cancer is crucial. Previous studies have demonstrated that age, sex, race, and histology play a role in bladder cancer metastasis[10]. However, no diagnostic and prognostic model studies are targeted exclusively at distant metastatic urothelial bladder cancer (DMUBC).

Nomograms are multivariable prediction models based on an individual’s characteristics. They are widely used in the cancer field as they can be used to predict individual patient risk and survival rates [11]. Meanwhile, Surveillance, Epidemiology, and End Results (SEER) database, (https://seer.cancer.gov/) is an authoritative source of population-based data, which records information about cancer incidence, stage, treatment, demographics, and survival[12]. Hence, our study aimed to construct nomograms of diagnostic and prognostic models based on UBC patients from the seer to evaluate related factors of distant metastases and cancer specific survival.

## Patients and methods

### Patients

Bladder cancer data were downloaded from the Incidence-SEER 18 Registries Research Plus Dataset (2000–2017) by SEER*Stat (version 8.3.9.2). Patients were included with the following criteria: (1) Bladder cancer was diagnosed with UBC (histology codes:8120-/-8122-/-8130-/-8131-); (2) available demographic variables included age, year of diagnosis, sex, and race; (3) available cancer-related clinical-pathological information included pathological tumor grade, tumor size, and TNM (Derived AJCC Stage Group, 6th ed. 2004_2015). Furthermore, patients who fulfilled the following criteria were excluded:(1) T0 (no evidence of primary tumor) and TX (not evaluable primary tumor); (2) NX (not evaluable regional lymph node metastasis); (3) MX (not evaluable distant metastasis) ;(4) tumor size was inaccurate or 0. Cancer distant metastasis diagnostic analysis was performed on all eligible patients. Subsequently, patients who reported available information, such as surgery, radiotherapy, chemotherapy, vital status, survival time, and cause-specific death classification (alive or dead due to cancer), were further selected for prognostic analysis. The diagnostic and prognostic cohorts were randomly divided into the training or validation groups using a 7:3 cut-off. The patients from the training group were used to construct nomograms, while the patients from the validation group were used to validate them.

One externally validated data was downloaded from the Incidence-SEER 17 Registries Research Plus Data (2000–2019). Patients were included based on previous criteria and a new TNM stage (Derived EOD, 2018+). The same classification methods were performed in the diagnostic and prognostic cohorts.

### Data collection and definitions

This study evaluated distant metastasis diagnostic predictors in UBC patients with the following variables: age, sex, race, grade, tumor size, T stage, N stage, and cancer histology type. Based on the above variables, surgery, radiotherapy, and chemotherapy were added to the prognostic model analysis. Age groups were divided into < 50 years ,50–59 years ,60–69 years, and > 69 years. Tumor size was categorized into four levels: <30 mm, 30–49mm, 50–99mm, and > 99 mm. T stage were classified as muscle invasion (T2/T3/T4) and non-muscle invasion (Tis/Ta/T1). M stages were also classified as non-distant metastasis (M0) and distant metastasis (M1). Surgery, chemotherapy, and radiation were categorized based on whether or not patient accepted the treatment. The primary outcome for prognostic model analysis was cancer-specific survival time (CSS), which was defined as the survival time between the months of initial diagnosis of UBC and cancer-specific death.

### Statistical analysis

R software (version 4.1.3) was used for the statistical analysis and original plot construction. All included patients were randomly separated into training and validation datasets using the “sample function” in R software. Fisher’s exact or Pearson Chi-square test analyzed the difference between the training and validation groups.

In the diagnostic cohort, a lasso regression analysis was conducted using the “glmnet” package, and significant variables were chosen as predictors. Based on important risk predictors, the “rms” package constructed a new diagnostic nomogram. Additionally, the receiver operating characteristic (ROC) curve and the corresponding area under the curve (AUC) were constructed to estimate the discrimination of the nomograms. A decision curve analysis (DCA) and calibration curves were also constructed to validate the predictive performance of the nomogram.

In the prognostic cohort, Cox-regression was performed on univariate to selected CSS-associated predictors in distant metastasis patients. Significant variables were then analyzed further in the forward multivariate Cox regression analysis to identify independent prognostic factors. Based on independent prognostic factors, a Cox-prognostic regression nomogram was constructed to predict the one-year, three-year, and five-year CSS. Similarly, ROC curve and AUC were constructed to estimate the discrimination performance of the nomogram. A DCA and calibration curves were also used to validate the predictive performance of the nomogram. Patients were categorized into low-risk and high-risk groups based on the nomogram’s median risk score, and survival outcomes were compared using Kaplan–Meier (K–M) survival curves. A two-sided P < 0.05 was considered a statistically significant difference.

To prove the accuracy and stability of diagnostic and prognostic nomograms, an externally validated dataset (2018–2019) was used to repeat above operations.

## Results

### Baseline characteristics of the study population

In the 2000–2017 dataset, 73,264 patients were pathologically diagnosed with UBC. Furthermore, 70% (51,284 cases) and 30% (21,980 cases) of patients were assigned to the training and validation groups, respectively. According to Table 1 and 2129 patients (2.9%) were diagnosed with distant metastasis. Grades IV was the most prevalent clinical grade (38.7% in the validation and 38.8% in the training sets). The most common T stage was non-muscle invasive bladder cancer Ta/Tis/T1 (72.7% in the validation and 72.5% in the training sets). In the histological type, papillary transitional cell carcinoma (8130) was detected in 72.5% of the training set and 72.6% of the validation set patients. Fisher’s exact and Pearson Chi-square test showed no significant difference between the training and validation sets (*P* > 0.05).

**Table 1:** Baseline clinical characteristics of urothelial bladder cancer patients

	Training group(*N* = 51,284)	Validation group (*N* = 21,980)	Overall (*N* = 73,264)	*X*^2^	*P*
Age, years				0.636	0.888
< 50	2303 (4.5%)	1016 (4.6%)	3319 (4.5%)		
50–59	6847 (13.4%)	2924 (13.3%)	9771 (13.3%)		
60–69	13,568 (26.5%)	5803 (26.4%)	19,371(26.4%)		
> 69	28,566 (55.7%)	12,237 (55.7%)	40,803(55.7%)		
Sex				0.512	0.474
Female	12,614 (24.6%)	5351 (24.3%)	17,965(24.5%)		
Male	38,670 (75.4%)	16,629 (75.7%)	55,299(75.5%)		
Race				4.588	0.205
American Indian/Alaska Native	180 (0.4%)	62 (0.3%)	242 (0.3%)		
Asian or Pacific Islander	2318 (4.5%)	954 (4.3%)	3272 (4.5%)		
Black	2729 (5.3%)	1130 (5.1%)	3859 (5.3%)		
White	46,057 (89.8%)	19,834 (90.2%)	65,891(89.9%)		
Grade				1.395	0.707
I	6511 (12.7%)	2821 (12.8%)	9332 (12.7%)		
II	14,738 (28.7%)	6245 (28.4%)	20,983(28.6%)		
III	10,123 (19.7%)	4398 (20.0%)	14,521(19.8%)		
IV	19,912 (38.8%)	8516 (38.7%)	28,428(38.8%)		
T				0.161	0.688
Ta/Tis/T1	37,188 (72.5%)	15,971 (72.7%)	53,159(72.6%)		
T2/T3/T4	14,096 (27.5%)	6009 (27.3%)	20,105(27.4%)		
*N*				1.042	0.791
N0	48,360 (94.3%)	20,701 (94.2%)	69,061(94.3%)		
N1	1445 (2.8%)	620 (2.8%)	2065 (2.8%)		
N2	1415 (2.8%)	634 (2.9%)	2049 (2.8%)		
N3	64 (0.1%)	25 (0.1%)	89 (0.1%)		
M				0.765	0.382
M0	49,775 (97.1%)	21,360 (97.2%)	71,135(97.1%)		
M1	1509 (2.9%)	620 (2.8%)	2129 (2.9%)		
Histologic Type (transitional cell cancer)				3.176	0.365
NOS (8120)	13,695 (26.7%)	5867 (26.7%)	19,562(26.7%)		
Spindle cells (8122)	255 (0.5%)	99 (0.5%)	354 (0.5%)		
Papillary (8130)	37,173 (72.5%)	15,960 (72.6%)	53,133(72.5%)		
Micropapillary (8131)	161 (0.3%)	54 (0.2%)	215 (0.3%)		
Tumor size, mm				0.599	0.897
<30	21,659 (42.2%)	9304 (42.3%)	30,963(42.3%)		
30–49	16,088 (31.4%)	6929 (31.5%)	23,017(31.4%)		
50–99	12,641 (24.6%)	5360 (24.4%)	18,001(24.6%)		
>99	896 (1.7%)	387 (1.8%)	1283 (1.8%)		

#### Incidence and risk factors of distant metastasis

**Fig. 1:**
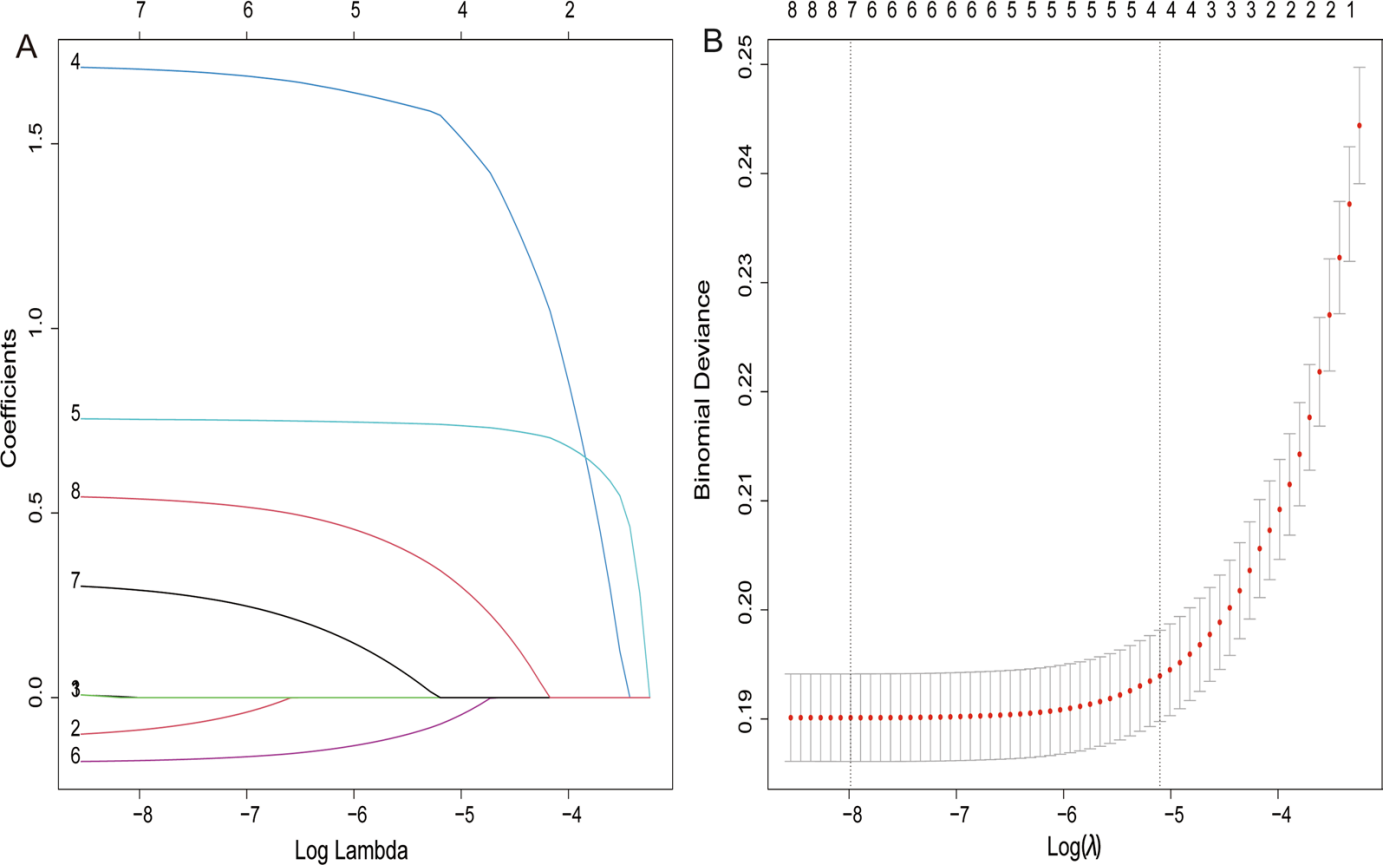
The lasso regression to evaluate the risk of distant metastasis in UBC patients

In initial diagnosis, 2129 patients (2.9%) had distant metastasis and 71,135 patients (97.1%) without it. The lasso regression analysis of eight potential variables revealed that distant metastasis in UBC patients might be predicted by the following four variables: tumor size, histologic type, T stage, and N stage (Fig. 1).

#### Diagnostic nomogram construction and validation

**Fig. 2:**
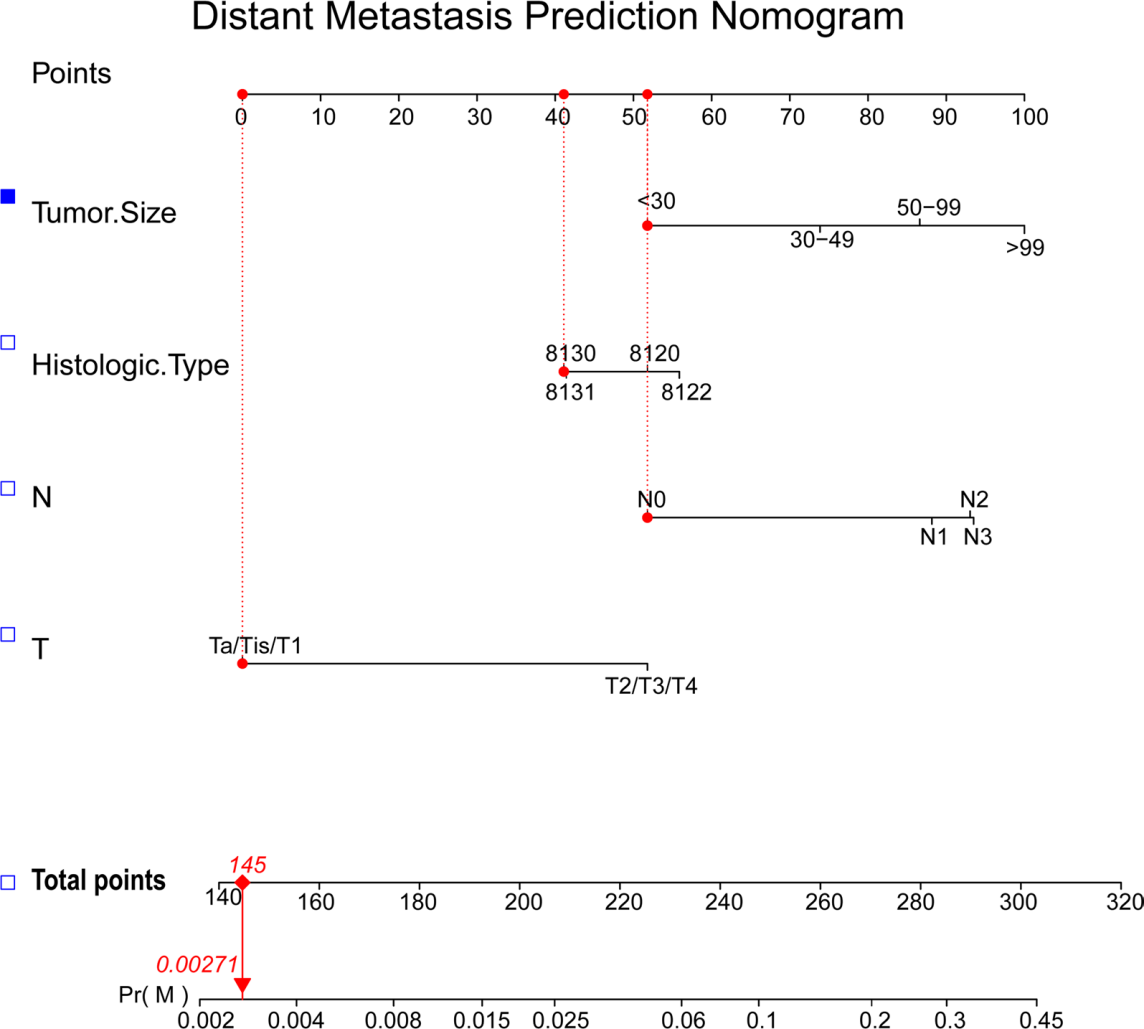
The nomogram to evaluate the risk of distant metastasis in UBC patients

**Fig. 3:**
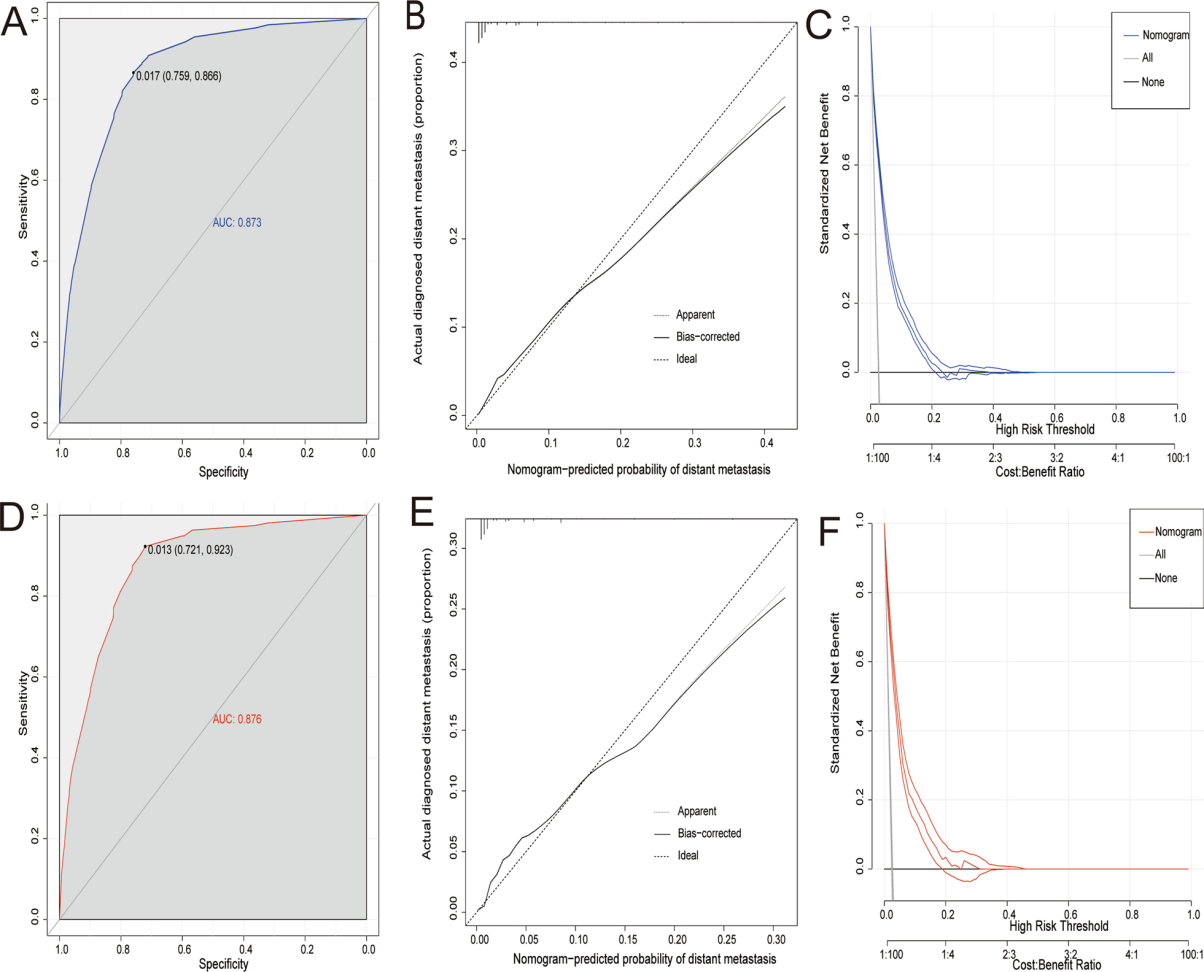
The receiver operating characteristic curve **A**, calibration curve **B**, and decision curve analysis **C** of the training set, and the receiver operating characteristic curve **D** calibration curve **E** and decision curve analysis **F** of the validation set

Based on the previous four important predictors, a novel nomogram was constructed to visually evaluate distant metastasis risk in UBC patients (Fig. 2). Subsequently, ROC curve was performed in the training and validation groups, with AUCs of 0.873 and 0.876, respectively (Fig. 3 A and, D). The calibration curves analysis showed a relatively high agreement between prediction and observation (Fig. 3B, and, E). As shown in DCA (Fig. 3 C and, F), the nomogram was an accurate and effective prediction tool of distant metastasis in UBC patients.

#### Prognostic factors for distant metastasis

In a study of 2129 distant metastasis patients, 1714 patients reported available information, which was used to analyze CSS prognostic factors. As indicated in Tables 1, 2 and 636 patients (95.4%) underwent surgery, 912(53.2%) underwent chemotherapy, and 378 (22.1%) received radiotherapy. The fisher’s exact and Chi-square tests showed no statistically significant differences between the training and validation groups. The univariate and multivariate COX regression analyses revealed that age, tumor size, surgery, and chemotherapy might be prognostic factors for distant metastasis patients (Table 3).

**Table 2:** Baseline clinical characteristics of patients diagnosed as urothelial bladder cancer with distant metastasis

	Training group(*N* = 1199)	Validation group(*N* = 515)	Overall (*N* = 1714)	*X*^2^	*P*
Age, years				2.442	0.486
< 50	49 (4.1%)	25 (4.9%)	74 (4.3%)		
50–59	190 (15.8%)	73 (14.2%)	263 (15.3%)		
60–69	303 (25.3%)	119 (23.1%)	422 (24.6%)		
> 69	657 (54.8%)	298 (57.9%)	955 (55.7%)		
Sex				0.057	0.811
Female	350 (29.2%)	154 (29.9%)	504 (29.4%)		
Male	966 (72.1%)	401 (69.7%)	1367 (71.4%)		
Race				Fisher	0.268
American Indian/Alaska Native	2 (0.2%)	1 (0.2%)	3 (0.2%)		
Asian or Pacific Islander	44 (3.7%)	29 (5.6%)	73 (4.3%)		
Black	99 (8.3%)	40 (7.8%)	139 (8.1%)		
White	1054 (87.9%)	445 (86.4%)	1499 (87.5%)		
Grade				Fisher	0.596
I	9 (0.8%)	3 (0.6%)	12 (0.7%)		
II	42 (3.5%)	12 (2.3%)	54 (3.2%)		
III	369 (30.8%)	155 (30.1%)	524 (30.6%)		
IV	779 (65.0%)	345 (67.0%)	1124 (65.6%)		
T				0.319	0.572
Ta/Tis/T1	181 (15.1%)	84 (16.3%)	265 (15.5%)		
T2/T3/T4	1018 (84.9%)	431 (83.7%)	1449 (84.5%)		
N				Fisher	0.670
N0	708 (59.0%)	303 (58.8%)	1011 (59.0%)		
N1	209 (17.4%)	100 (19.4%)	309 (18.0%)		
N2	272 (22.7%)	107 (20.8%)	379 (22.1%)		
N3	10 (0.8%)	5 (1.0%)	15 (0.9%)		
Histologic type (transitional cell cancer)				Fisher	0.593
NOS (8120)	782 (65.2%)	321 (62.3%)	1103 (64.4%)		
Spindle cells (8122)	23 (1.9%)	8 (1.6%)	31 (1.8%)		
Papillary (8130)	386 (32.2%)	183 (35.5%)	569 (33.2%)		
Micropapillary (8131)	8 (0.7%)	3 (0.6%)	11 (0.6%)		
Tumor size, mm				1.259	0.739
<30	151 (12.6%)	73 (14.2%)	224 (13.1%)		
30–49	354 (29.5%)	153 (29.7%)	507 (29.6%)		
50–99	610 (50.9%)	258 (50.1%)	868 (50.6%)		
>99	84 (7.0%)	31 (6.0%)	115 (6.7%)		
Surgery				< 0.01	1
No	55 (4.6%)	23 (4.5%)	78 (4.6%)		
Yes	1144 (95.4%)	492 (95.5%)	1636 (95.4%)		
Chemotherapy				0.475	0.491
No	554 (46.2%)	248 (48.2%)	802 (46.8%)		
Yes	645 (53.8%)	267 (51.8%)	912 (53.2%)		
Radiotherapy				0.153	0.696
No	931 (77.6%)	459 (79.8%)	1516 (79.2%)		
Yes	268 (22.4%)	110 (21.4%)	378 (22.1%)		

**Table 3:** Univariate and multivariate Cox analyses in urothelial bladder cancer patients with distant metastasis

	Univariate analysis			Multivariate analysis		
	HR	95%CI	*P*	HR	95%CI	*P*
Age, years						
< 50	Reference			Reference		
50–59	1.328	0.954–1.848	0.093	1.221	0.875–1.703	0.241
60–69	1.224	0.890–1.684	0.214	1.207	0.876–1.664	0.250
> 69	1.869	1.375–2.538	< 0.001	1.207 1.607	1.178–2.191	0.003
Sex						
Female	Reference					
Male	0.910	0.800-1.035	0.151			
Race						
American Indian/Alaska Native	Reference					
Asian or Pacific Islander	1.279	0.309–5.293	0.735			
Black	0.979	0.241–3.977	0.976			
White	1.204	0.301–4.822	0.793			
Grade						
I	Reference					
II	0.785	0.379–1.623	0.513			
III	0.859	0.443–1.666	0.654			
IV	0.841	0.436–1.623	0.605			
T						
Ta/Tis/T1	1.062	0.901–1.250	0.474			
T2/T3/T4	Reference					
N						
N0	Reference			Reference		
N1	0.889	0.758–1.043	0.149	1.053	0.895–1.239	0.534
N2	0.860	0.743–0.995	0.043	0.990	0.852–1.151	0.897
N3	1.543	0.825–2.883	0.174	1.783	0.948–3.351	0.072
Histologic type (transitional cell cancer)						
NOS (8120)	Reference			Reference		
Spindle cells (8122)	1.744	1.151–2.643	0.009	1.426	0.938–2.168	0.096
Papillary (8130)	0.818	0.721–0.929	0.002	0.783	0.688–0.891	< 0.001
Micropapillary (8131)	0.643	0.288–1.435	0.281	1.052	0.469–2.362	0.901
Tumor size, mm						
<30	Reference			Reference		
30–49	1.250	1.021–1.530	0.031	1.319	1.075–1.620	0.008
50–99	1.627	1.346–1.966	< 0.001	1.668	1.377–2.021	< 0.001
>99	1.570	1.185–2.081	0.002	1.632	1.225–2.174	< 0.001
Surgery						
No	Reference			Reference		
Yes	0.617	0.468–0.814	< 0.001	0.570	0.429–0.755	< 0.001
Chemotherapy						
No	Reference			Reference		
Yes	0.382	0.339–0.431	< 0.001	0.383	0.338–0.434	< 0.001
Radiotherapy						
No	Reference			Reference		
Yes	1.172	1.019–1.347	0.026	1.218	1.056–1.405	0.007

#### Prognostic nomogram construction and validation

**Fig. 4:**
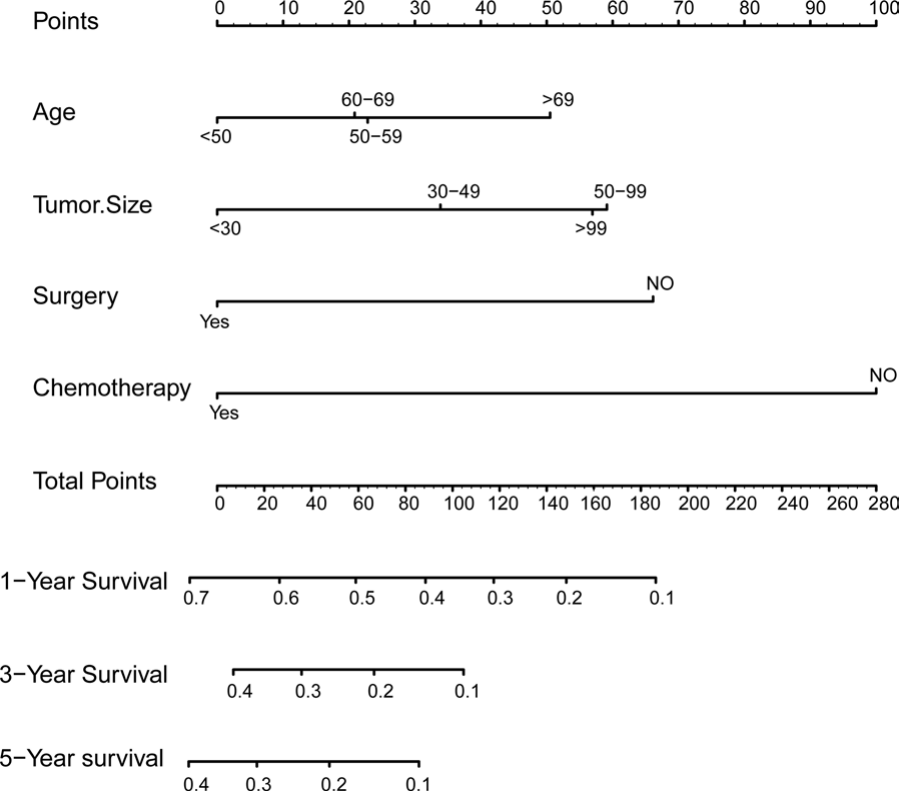
A nomogram to predictive the CSS of UBC patients with distant metastasis for the 1, 3, and 5 years

**Fig. 5:**
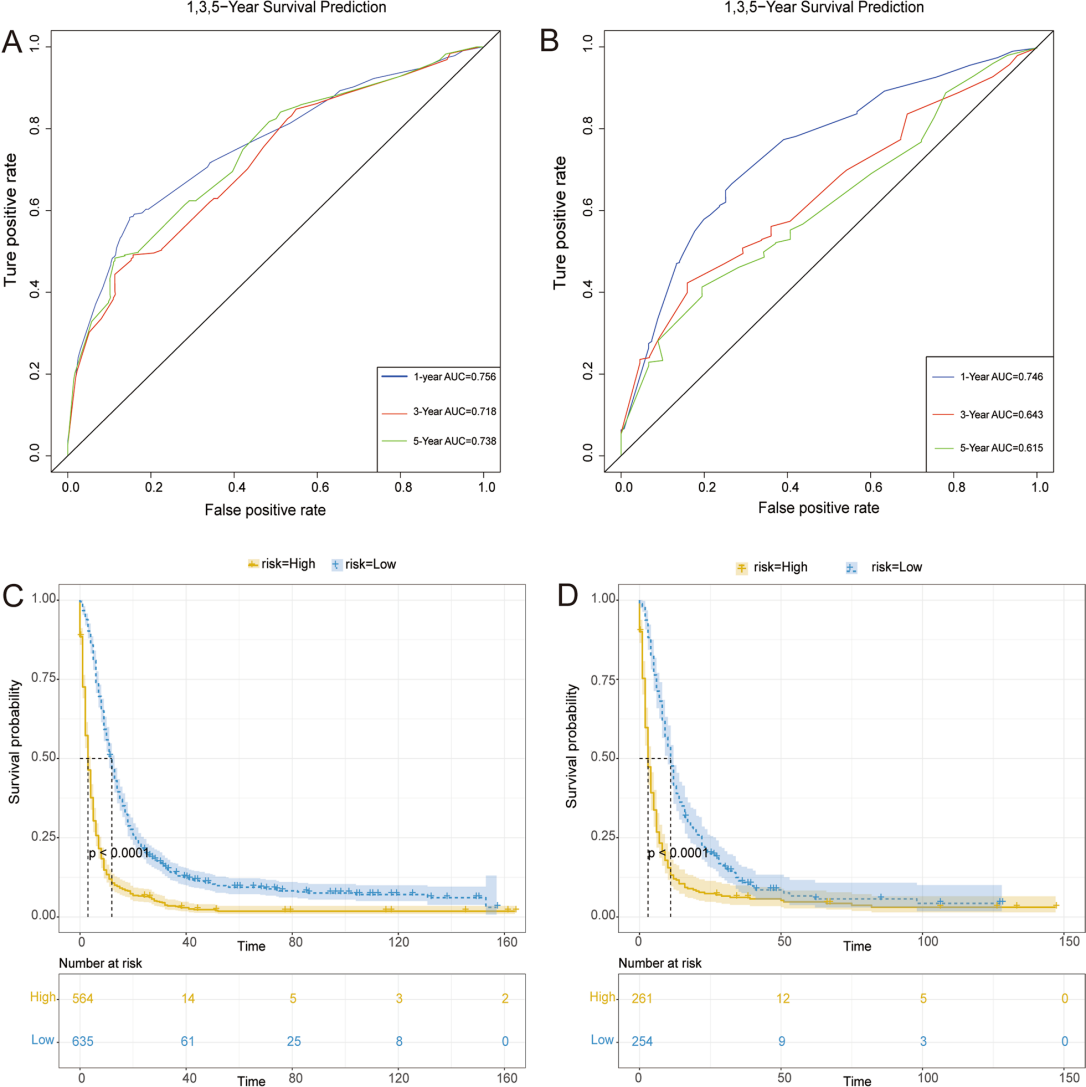
Time-dependent ROC curve analysis of the nomogram for the 1, 3, and 5 years in the training set (**A**) and the validation set (**B**). The Kaplan–Meier survival curves of the patients in the training set (**C**) and in the validation set (**D**)

**Fig. 6:**
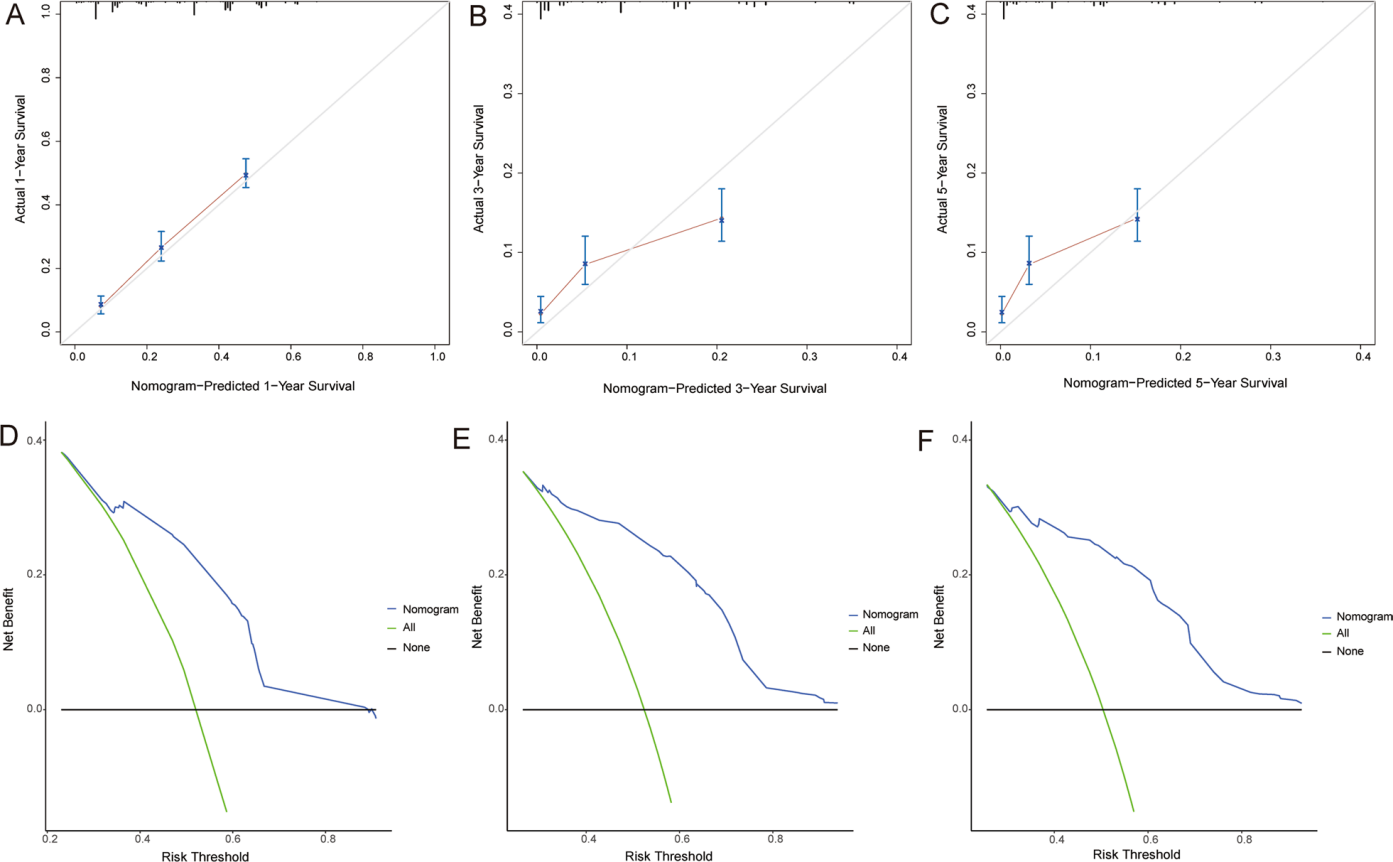
The calibration curves of the nomogram for the 1 (**A**), 3 (**B**), and 5 years (**C**) in the training set. The decision curve analysis of the nomogram for the1 (**D**),3 (**E**), and 5 years (**F**) in the training set

**Fig. 7:**
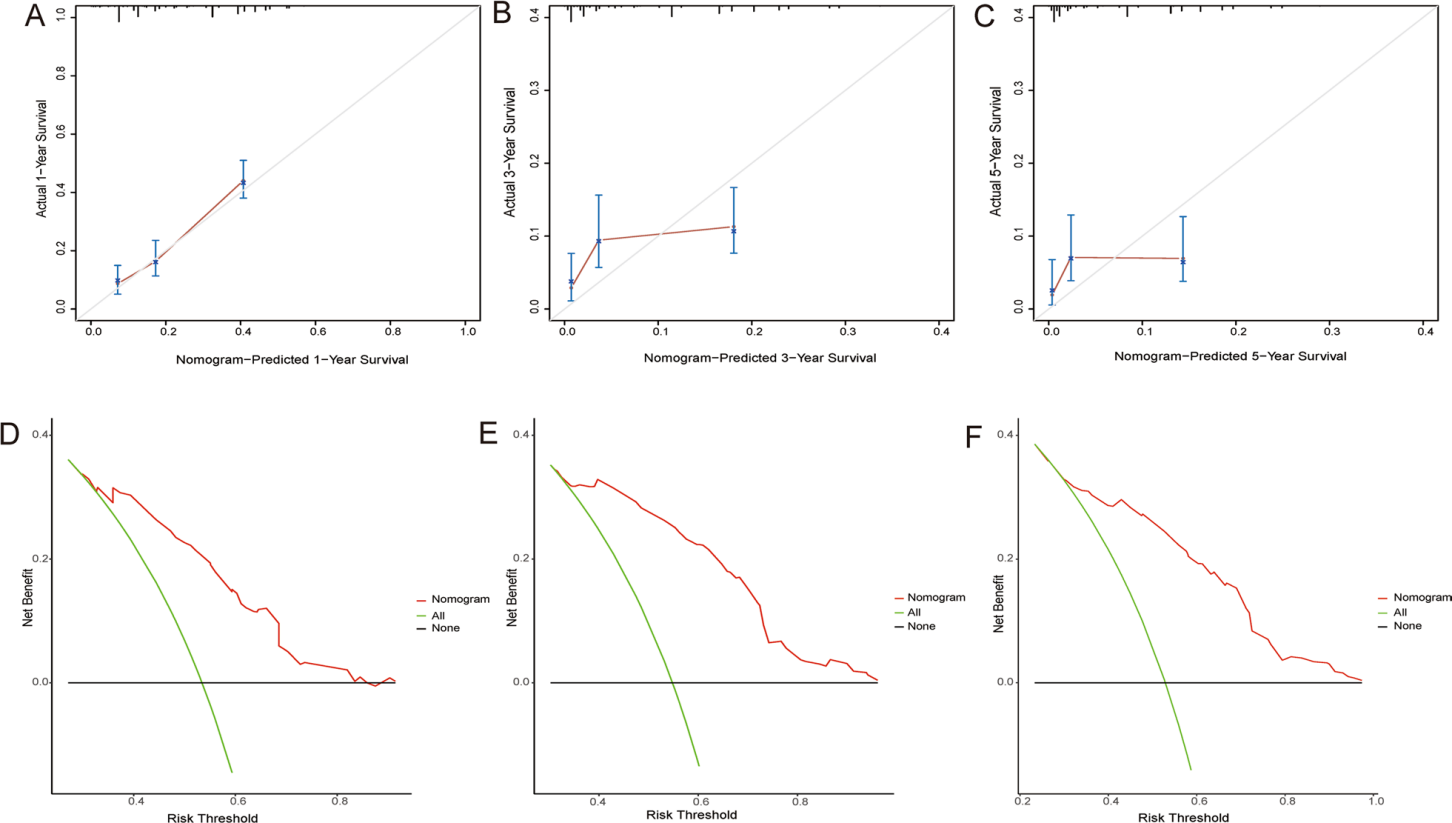
The calibration curves of the nomogram for the 1 (**A**), 3 (**B**), and 5 years (**C**) in the validation set. The decision curve analysis of the nomogram for the 1 (**D**), 3 (**E**), and 5 years (**F**) in the validation set

A novel nomogram was constructed based on the four prognostic factors (Fig. 4). According to time-dependent ROC curves, AUCs in the training group for 1-, 3-, and 5-years CSS were 0.756, 0.718, and 0.738, respectively (Fig. 5 A). In the validation group, 0.746, 0.643, and 0.615 were the values of AUCs 1-,3-,5-years CSS, respectively (Fig. 5B). In the training and validation groups, patients were divided into high-risk (564 vs. 261) and low-risk (635 vs. 254) groups, respectively. According to the K–M survival analysis, high-risk group patients had a lower rate of CSS (*P* < 0.05) than low-risk group patients (Fig. 5 C, D). In addition, each calibration curves of 1-, 3-, and 5-years CSS shown a good correlation between actual observation and nomogram prediction in training (Fig. 6 A–C) and validation groups (Fig. 7 A–C). DCA results showed the nomogram with an effective and auxiliary value in clinical practice (Fig. 6D–F and, 7D–F).

#### Validation in external dataset

**Fig. 8:**
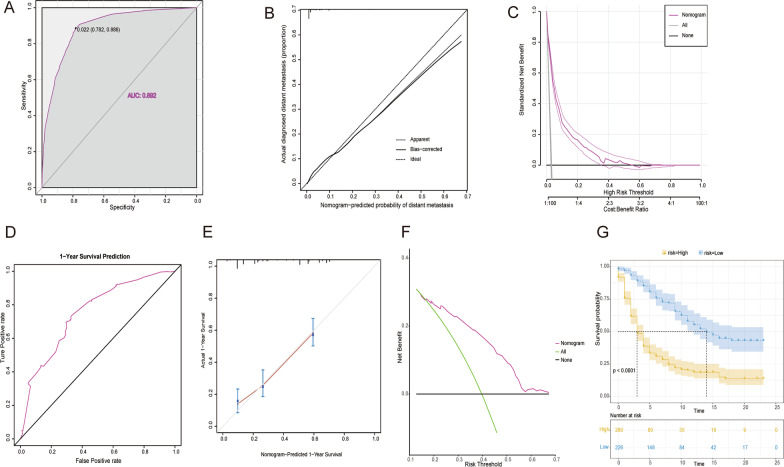
The receiver operating characteristic curve **A**, calibration curve **B**, and decision curve analysis **C** of external dataset. The receiver operating characteristic curve **D**, calibration curve **E**, and decision curve analysis **F** of 1year cancer-specific survival. The Kaplan–Meier survival curves of the patients in external dataset **G**

In the diagnostic model, 17,839 patients were diagnosed with UBC between 2018 and 2019, and 536 (3%) with distant metastasis. Based on the above diagnostic nomogram, ROC of external dataset showed high accuracy in diagnosis distant metastasis (AUC: 0.892, Fig. 8 A). The calibration curves analysis also revealed a relatively high agreement between the prediction and observation (Fig. 8B). DCA result suggested that the diagnostic nomogram also aids in clinical practice (Fig. 8 C).

In the prognostic model, 506 eligible distant metastasis patients reported available treatment and prognosis information. Notably, only a one-year survival prognosis analysis can be performed due to all follow-ups of less than three years. The time-dependent ROC curve accurately predicted one-year survival patients using the prognostic nomogram (Fig. 8D). Furthermore, calibration curves of the one-years CSS revealed a strong correlation between nomogram prediction and actual observation (Fig. 8E). DCA results have also demonstrated an effective and auxiliary value in clinical practice (Fig. 8 F). Furthermore, K–M survival analysis showed that the nomogram can significantly discriminate between low-risk group patients and high-risk patients, who had a lower CSS rate compared to low-risk group patients (*P* < 0.05) (Fig. 8G).

## Discussion

This study investigated the risk and prognostic factors of distant metastasis UBC patients. The diagnostic model found that tumor size, histologic type, N stage and T stage were all important predictive factors. Our findings are partially different from those of previous studies. Wang et al. analyzed bladder cancer patients and reported that patients of 40–60 years, female and black race, were more common in the distant metastasis group[13]. Simultaneously, Shou et al. performed a SEER-based study and found that bladder cancer patients with high tumor stage, positive lymph node metastasis, and advanced histologic grade were more likely to have distant metastasis [14]. However, the above two studies included all types of bladder cancer variants and only conducted a univariate analysis to find a group difference between distant metastasis and non- distant metastasis groups. According to previous studies, a higher tumor stage indicates a lower differentiation degree in tumor tissues, reducing cellular adhesion ability between poorly differentiated tumor cells and contributed to metastasis[15]. However, our result showed that tumor grade was not a predictive factor in distant metastasis. This may be related to the fact that bladder cancer commonly metastasizes in lymph nodes before spreading to distant organs. In addition, we used Lasso regression analysis to select variables that predicted distant shifts, allowing us to screen out more predictive variables. Consequently, lymph node positivity, tumor size, pathological classification, and T-stage were more predictive of distant metastasis relative to tumor grade. In addition, women are more likely to be diagnosed with bladder cancer at an advanced stage due to late presentation, but no correlation was found with distant metastasis [16]. With a mean age of 70–84 years at the time of diagnosis and 84.5% of patients older than 60 years in our study, advanced age is a common factor for bladder cancer, which may be associated with long-term smoking exposure and decreased DNA repair[17]. The correlation between distant metastasis and advanced age has not been further studied or reported. The clinical T stage was used to evaluate the tumor burden, with T2 muscle infiltration bladder cancer usually having a 25% rate of positive lymph node metastasis, which is the primary mode of metastasis in various stages of bladder cancer[18]. Furthermore, Tian et al. excluded distant metastasis patients and found that T stage, tumor grade, age, and tumor size were independent risk factors for lymph node metastasis in UBC, suggesting a correlation between tumor size and lymph node metastasis [19]. In addition, clinical prediction studies for distant metastasis in UBC are currently limited. Our distant metastasis prediction models from four important variables may benefit patients’ management and clinicians’ decision*-*making.

After univariate and multivariate Cox regression analysis, the prognostic model found that age, tumor size, surgery and chemotherapy were independent prognosticators for DMUBC patients. Similar results have been reported in previous studies. Based on the bladder cancer seer database, Wang et al. found that age, tissue type, chemotherapy, and surgery were independent prognosticators for distant metastasis [13]. However, the above study acted overall survival as the primary outcome, and all types of bladder cancer were included. In contrast, our study used CSS as the outcome and only included DMUBC, which may be the main reason for the different results. Asimakopoulos et al. performed a retrospective study and developed a prognostic nomogram that confirmed T1 substaging, tumor size and tumor location as independent prognosticators of five-years disease-free survival in T1 bladder cancer patients[20]. Additionally, Tian et al. included TCBC patients from SEER database. The result found that age, race, tumor size, tumor stage, T stage, and N stage were independent prognostic factors for overall survival in patients with positive lymph node metastases [19]. Similarly, tumor size has been considered as a prognostic factor for metastatic and non-metastatic bladder tumors. In our study, tumor grade and T-stage were not used as prognostic factors for patients with distant metastases. From Table 2, we found that most patients with distant metastases had advanced bladder muscle infiltration and high tumor stage, which may lead to bias as prognostic factors for tumor-specific survival. In contrast, chemotherapy is now used as a first-line treatment for patients with distant metastases. Surgery and chemotherapy play an important role in the current treatment of metastatic bladder cancer. In 2017, Abufaraj et al. published a systematic review in the European urology journal and found that surgical resection can achieve long-term tumor control in some metastatic bladder cancer patients[21]. Similar to the results of previous studies, our study suggests that surgery and chemotherapy can act as independent prognosticators and effectively prolong survival time for DMUBC.

Recently, molecular biology and genetic technology have played an important role in the diagnosis and prognosis of metastasis bladder cancer. Highly expressed CD164, CD133^+^, CD24^+^, and cafs genes were associated with metastasis and invasion of bladder cancer[22–24]. Overexpression of SOX30 inhibits the proliferation, invasion, and migration of bladder cancer[25]. Although, the molecules and genes mentioned above can accurately predict bladder cancer metastasis and prognosis; a simple and effective clinical assessment modality was required. The current study has a number of advantages. First, our data are based on an authoritative SEER database, which supplied large samples of UBC. Second, this is the first univariate and multivariate research to analyze the prognostic factors in DMUBC. Third, we constructed two nomograms based on the results, which might assist in clinical decision-making and patient management.

However, our study also had certain limitations. To begin, SEER offers limited information on treatment regimens, such as the details of adjuvant chemotherapy and surgery for distant metastasis patients. Then, all included samples are bladder cancer patients who had distant metastasis at the time of diagnosis, but not patients who developed distant metastasis later on. Lastly, the study was retrospective, and there may have been some selection bias.

## Conclusion

Our study revealed that tumor size, histologic type, N stage and T stage were important risk predictors of distant metastasis in urothelial bladder cancer patients. Furthermore, age, tumor size, chemotherapy, and surgery were all independent prognostic factors for urothelial bladder cancer patients with distant metastasis. The two nomograms could effectively predict the occurrence and prognosis risk of urothelial bladder cancer patients with distant metastasis.

## Data Availability

The dataset in this study were download from the SEER dataset (https://seer.cancer.gov/).
